# Health Center Testing for SARS-CoV-2 During the COVID-19 Pandemic — United States, June 5–October 2, 2020

**DOI:** 10.15585/mmwr.mm6950a3

**Published:** 2020-12-18

**Authors:** Lisa Romero, Leah Zilversmit Pao, Hollie Clark, Catharine Riley, Sharifa Merali, Michael Park, Carrie Eggers, Stephanie Campbell, Cuong Bui, Joshua Bolton, Xuan Le, Robyn Neblett Fanfair, Michelle Rose, Alison Hinckley, Charlene Siza

**Affiliations:** ^1^CDC COVID-19 Response Team; ^2^Bureau of Primary Heath Care, Health Resources and Services Administration, Rockville, Maryland.

Long-standing social inequities and health disparities have resulted in increased risk for coronavirus disease 2019 (COVID-19) infection, severe illness, and death among racial and ethnic minority populations. The Health Resources and Services Administration (HRSA) Health Center Program supports nearly 1,400 health centers that provide comprehensive primary health care* to approximately 30 million patients in 13,000 service sites across the United States.^†^ In 2019, 63% of HRSA health center patients who reported race and ethnicity identified as members of racial ethnic minority populations (*1*). Historically underserved communities and populations served by health centers have a need for access to important information and resources for preventing exposure to SARS-CoV-2, the virus that causes COVID-19, to testing for those at risk, and to follow-up services for those with positive test results.^§^ During the COVID-19 public health emergency, health centers^¶^ have provided and continue to provide testing and follow-up care to medically underserved populations**; these centers are capable of reaching areas disproportionately affected by the pandemic.^††^ HRSA administers a weekly, voluntary Health Center COVID-19 Survey^§§^ to track health center COVID-19 testing capacity and the impact of COVID-19 on operations, patients, and personnel. Potential respondents can include up to 1,382 HRSA-funded health centers.^¶¶^ To assess health centers’ capacity to reach racial and ethnic minority groups at increased risk for COVID-19 and to provide access to testing, CDC and HRSA analyzed survey data for the weeks June 5–October 2, 2020*** to describe all patients tested (3,194,838) and those who received positive SARS-CoV-2 test results (308,780) by race/ethnicity and state of residence. Among persons with known race/ethnicity who received testing (2,506,935), 36% were Hispanic/Latino (Hispanic), 38% were non-Hispanic White (White), and 20% were non-Hispanic Black (Black); among those with known race/ethnicity with positive test results, 56% were Hispanic, 24% were White, and 15% were Black. Improving health centers’ ability to reach groups at increased risk for COVID-19 might reduce transmission by identifying cases and supporting contact tracing and isolation. Efforts to improve coordination of COVID-19 response-related activities between state and local public health departments and HRSA-funded health centers can increase access to testing and follow-up care for populations at increased risk for COVID-19.

HRSA administers a weekly voluntary Health Center COVID-19 Survey to track health center COVID-19 testing capacity and the impact of COVID-19 on operations, patients, and staff members. The 1,382 health centers asked to complete the survey are located in all 50 states, the District of Columbia (DC), and five territories and freely associated states.^†††^ This analysis used survey data from the weeks ending June 5–October 2, 2020, to describe the patient population and, among all patients who received testing for SARS-CoV-2 with viral tests (i.e., polymerase chain reaction and antigen tests), the numbers and proportions of persons with tests and positive results by race/ethnicity and state of residence. State survey response rates ranged from 68% to 80% among health centers. Proportions of patients receiving SARS-CoV-2 tests and positive test results included unreported race/ethnicity as a separate category.

As reported in the HRSA Uniform Data System in 2019, HRSA-funded health centers reported that 35% of their national patient population was White, 35% Hispanic,^§§§^ 18% Black, 4% Asian, 1% American Indian/Alaska Native (AI/AN), 1% Native Hawaiian/Other Pacific Islander, and 1.3% multiracial persons; race/ethnicity was not reported for 6% of the patient population (Figure) (*1*). By comparison, the 2019 American Community Survey^¶¶¶^ estimated that the U.S. population comprises 60% White, 18% Hispanic, 12% Black, 6% Asian, 1% AI/AN, 0.2% Native Hawaiian/Other Pacific Islander, and 3% multiracial persons.

**FIGURE:**
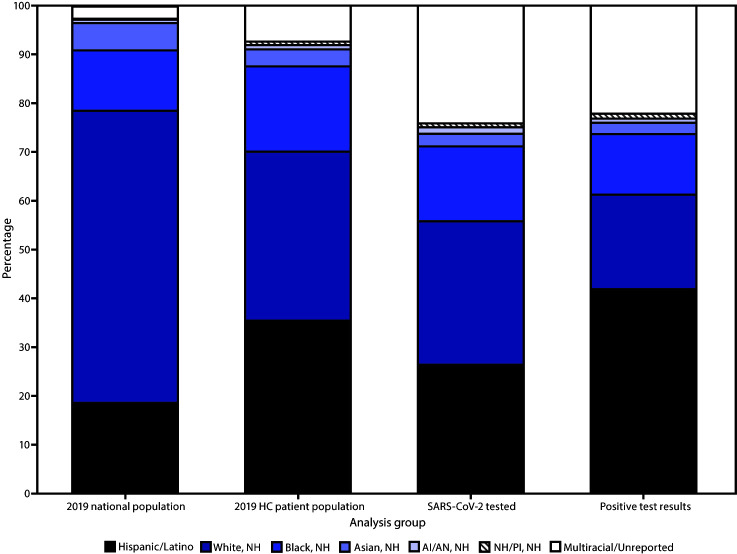
Racial/ethnic distribution of 2019 national* and Health Resources and Services Administration (HRSA)–funded health center^†^ patient populations^§^ and persons who received testing and had positive SARS-CoV-2 test results^¶^ — Health Center COVID-19 Survey, United States, June 5–October 2, 2020 **Abbreviations:** AI/AN = American Indian/Alaska Native; COVID-19 = coronavirus disease 2019; HC = health center; NH/PI = Native Hawaiian/Other Pacific Islander; NH = non-Hispanic. * Data from the 2019 American Community Survey (https://data.census.gov/cedsci/table?d=ACS%201-Year%20Estimates%20Data%20Profiles&tid=ACSDP1Y2019. DP05&hidePreview=false). Data include non-Hispanic NH/PI (0.2%), not visible in figure and do not include other race (0.3%). Persons with multiracial or unreported race/ethnicity have an unreported or non-Hispanic ethnicity. ^†^ HRSA–funded health centers include both Federally Qualified Health Centers (FQHCs) and Health Center Program Look-Alikes (i.e, meets all Health Care Center Program requirements but does not receive federal funding). During the COVID-19 pandemic, HRSA provided one-time COVID-19 funding to FQHCs and Health Center Look-Alikes to purchase, administer, and expand capacity for testing to monitor and suppress COVID-19 testing and response-related activities. ^§^ HRSA 2019 Uniform Data System. https://data.hrsa.gov/tools/data-reporting/program-data/national. ^¶^ HRSA COVID-19 Survey, June 5–October 2, 2020. Data for the number tested or the number tested positive are aggregated by health centers before submission and cannot be deduplicated, which might inflate or misrepresent the number of patients tested or who had positive test results.

During June 5–October 2, 2020, health centers responding to the survey reported that 3,194,838 patients received testing and 308,780 had positive SARS-CoV-2 test results. Compared to other jurisdictions, Texas reported the highest number of patients who received testing (353,081; 11%), and California reported the highest number of patients who had positive test results (46,113; 15%). Based on data reported to the Health Center COVID-19 Survey, White and Hispanic patients each accounted for 29% of patients who received testing for SARS-CoV-2 (Table 1) and, among patients who received positive test results, 19% were White and 45% were Hispanic (Table 2). Overall, race was not reported for 22% (687,903) of patients tested and 19% of patients with positive test results (57,208); 1% (26,386) of patients receiving testing and 1% (2,378) of patients with positive test results were multiracial. In Puerto Rico, 96% of patients receiving testing were Hispanic; among other jurisdictions, the highest proportions of patients receiving testing who were Hispanic were in Nevada (9,990; 56%) and New Mexico (11,705; 56%). Compared with all other jurisdictions, California reported the most Hispanic patients who received testing (186,034) and the most positive test results among Hispanic patients (33,310; 18%). Among those with positive test results, Puerto Rico reported the largest proportion of Hispanic patients (2,095; 98%) and New Mexico the second highest proportion (531; 73%). Nationally, Black patients accounted for 15% of patients receiving testing and 12% of those who received positive test results. Mississippi reported the highest proportion of patients who received testing who were Black (25,850; 67%) and the highest proportion of those who had positive test results who were Black (2,348; 69%). Georgia reported the largest number of tests conducted (42,889; 43%) and positive test results (4,204; 32%) among Black patients.

**TABLE 1:** Viral testing for SARS-CoV-2,* by race/ethnicity and jurisdiction^†^ — Health Center COVID-19 Survey, United States, June 5–October 2, 2020

Jurisdiction	No.^§^ of FQHCs^¶^	Response range** (%)	Total no. patients tested^††^	Race/Ethnicity, no. tested (row %)							
				Hispanic/Latino	White, NH	Black, NH	Asian, NH	AI/AN, NH	NH/PI, NH	Multiracial	Unreported
**United States**	**1,382–1,376**	**68–80**	**3,194,838**	**913,718 (29)**	**941,017 (29)**	**491,311 (15)**	**77,528 (2)**	**36,837 (1)**	**5,161 (—)**	**26,386 (1)**	**687,903 (22)**
Alabama	17	59–94	46,146	3,393 (7)	16,089 (35)	21,262 (46)	183 (—)	139 (—)	4 (—)	241 (1)	4,800 (10)
Alaska	27	44–74	57,147	1,788 (3)	11,510 (20)	528 (1)	967 (2)	20,785 (36)	83 (—)	362 (1)	21,080 (37)
American Samoa	1	0–100	1,567	104 (7)	46 (3)	0 (—)	55 (4)	12 (1)	0 (—)	2 (—)	8 (1)
Arizona	23	65–83	53,455	27,220 (51)	15,584 (29)	1,509 (3)	335 (1)	1,741 (3)	50 (—)	392 (1)	6,573 (12)
Arkansas	12	50–100	51,488	7,855 (15)	24,988 (49)	13,061 (25)	243 (—)	152 (—)	21 (—)	189 (—)	4,169 (8)
California	175–178	59–74	336,454	186,034 (55)	51,114 (15)	26,371 (8)	20,103 (6)	949 (—)	634 (—)	4,891 (1)	45,217 (13)
Colorado	19	68–95	59,401	24,702 (42)	19,064 (32)	5,165 (9)	1,166 (2)	324 (1)	31 (—)	791 (1)	8,123 (14)
Connecticut	16	56–94	83,507	27,872 (33)	15,803 (19)	8,258 (10)	1,190 (1)	218 (—)	20 (—)	349 (—)	29,613 (35)
Delaware	3	33–100	2,356	894 (38)	466 (20)	850 (36)	31 (1)	9 (—)	4 (—)	14 (1)	88 (4)
District of Columbia	8	63–100	18,438	5,632 (31)	1,628 (9)	8,554 (46)	274 (1)	30 (—)	2 (—)	89 (—)	2,215 (12)
Federated States of Micronesia	4	25–100	198	0 (—)	0 (—)	0 (—)	54 (27)	0 (—)	0 (—)	0 (—)	0 (—)
Florida	47	62–87	257,119	97,542 (38)	51,704 (20)	38,483 (15)	1,868 (1)	189 (—)	44 (—)	2,431 (1)	64,665 (25)
Georgia	35	60–91	100,909	15,410 (15)	32,664 (32)	42,889 (43)	1,119 (1)	67 (—)	11 (—)	862 (1)	7,861 (8)
Guam	1	0–100	8,574	31 (—)	199 (2)	38 (—)	2,572 (30)	9 (—)	4 (—)	432 (5)	268 (3)
Hawaii	14	36–86	8,894	850 (10)	911 (10)	91 (1)	1,325 (15)	13 (—)	2,833 (32)	286 (3)	1,081 (12)
Idaho	14	71–100	12,111	3,014 (25)	6,281 (52)	168 (1)	100 (1)	1,028 (8)	25 (—)	26 (—)	1,460 (12)
Illinois	45	64–82	162,663	51,534 (32)	37,781 (23)	22,276 (14)	3,097 (2)	217 (—)	44 (—)	676 (—)	46,966 (29)
Indiana	27	48–78	20,639	6,031 (29)	5,570 (27)	2,035 (10)	967 (5)	34 (—)	3 (—)	112 (1)	5,876 (28)
Iowa	14	64–93	30,891	5,292 (17)	14,786 (48)	1,511 (5)	447 (1)	222 (1)	16 (—)	235 (1)	8,357 (27)
Kansas	19	53–100	25,472	6,582 (26)	13,631 (54)	1,972 (8)	326 (1)	336 (1)	93 (—)	212 (1)	2,264 (9)
Kentucky	25	76–96	64,494	3,453 (5)	51,839 (80)	4,141 (6)	446 (1)	36 (—)	15 (—)	506 (1)	4,017 (6)
Louisiana	36	61–81	48,007	5,297 (11)	16,396 (34)	21,333 (44)	487 (1)	143 (—)	39 (—)	323 (1)	3,968 (8)
Maine	18	50–89	9,049	600 (7)	6,614 (73)	515 (6)	40 (—)	40 (—)	7 (—)	43 (—)	1,189 (13)
Marshall Islands	1	0–100	121	0 (—)	0 (—)	0 (—)	1 (1)	0 (—)	0 (—)	0 (—)	0 (—)
Maryland	17	47–82	8,898	2,696 (30)	1,824 (20)	2,629 (30)	117 (1)	248 (3)	3 (—)	135 (2)	1,242 (14)
Massachusetts	37–38	59–89	153,411	51,639 (34)	52,813 (34)	16,444 (11)	5,326 (3)	181 (—)	61 (—)	817 (1)	25,712 (17)
Michigan	39	51–79	99,960	12,870 (13)	53,804 (54)	13,622 (14)	1,799 (2)	305 (—)	46 (—)	1,040 (1)	16,412 (16)
Minnesota	16	56–81	16,645	2,828 (17)	5,093 (31)	3,627 (22)	1,178 (7)	794 (5)	5 (—)	32 (—)	3,077 (18)
Mississippi	20	65–95	38,843	3,411 (9)	8,300 (21)	25,850 (67)	218 (1)	59 (—)	9 (—)	326 (1)	605 (2)
Missouri	28–29	66–90	78,075	7,414 (9)	36,275 (46)	16,802 (22)	677 (1)	207 (—)	57 (—)	567 (1)	15,895 (20)
Montana	14	43–86	18,377	368 (2)	7,522 (41)	31 (—)	18 (—)	441 (2)	7 (—)	60 (—)	9,928 (54)
Nebraska	7	57–86	9,224	3,585 (39)	2,167 (23)	2,008 (22)	896 (10)	40 (—)	6 (—)	97 (1)	422 (5)
Nevada	8	38–88	17,827	9,990 (56)	4,102 (23)	938 (5)	1,145 (6)	47 (—)	157 (1)	94 (1)	1,322 (7)
New Hampshire	10	70–100	3,535	936 (26)	2,140 (61)	88 (2)	80 (2)	6 (—)	3 (—)	10 (—)	272 (8)
New Jersey	23–24	33–67	51,393	26,433 (51)	9,698 (19)	7,173 (14)	603 (1)	62 (—)	43 (—)	398 (1)	6,887 (13)
New Mexico	16	75–100	20,857	11,705 (56)	4,115 (20)	295 (1)	98 (—)	1,427 (7)	12 (—)	208 (1)	2,985 (14)
New York	63	48–67	204,075	31,877 (16)	32,798 (16)	24,734 (12)	5,972 (3)	194 (—)	30 (—)	1,606 (1)	106,749 (52)
North Carolina	39	46–69	65,685	14,505 (22)	18,118 (28)	22,130 (34)	661 (1)	524 (1)	66 (—)	334 (1)	9,326 (14)
North Dakota	4	75–100	5,003	199 (4)	2,425 (48)	385 (8)	170 (3)	300 (6)	9 (—)	35 (1)	1,476 (30)
Northern Mariana Islands	1	0–100	0	0 (—)	0 (—)	0 (—)	0 (—)	0 (—)	0 (—)	0 (—)	0 (—)
Ohio	51	67–82	72,400	7,604 (11)	37,561 (52)	13,461 (19)	1,216 (2)	443 (1)	39 (—)	550 (1)	11,418 (16)
Oklahoma	21	52–95	13,147	2,833 (22)	5,284 (40)	1,151 (9)	86 (1)	480 (4)	16 (—)	145 (1)	3,117 (24)
Oregon	30	73–90	17,782	6,771 (38)	7,471 (42)	474 (3)	400 (2)	686 (4)	40 (—)	145 (1)	1,697 (10)
Palau	1	0–100	1,612	0 (—)	254 (16)	4 (—)	334 (21)	0 (—)	0 (—)	0 (—)	0 (—)
Pennsylvania	43	67–91	54,143	9,563 (18)	22,464 (41)	13,911 (26)	2,549 (5)	118 (—)	13 (—)	651 (1)	4,745 (9)
Puerto Rico	21–22	73–91	28,909	27,709 (96)	134 (—)	5 (—)	0 (—)	3 (—)	0 (—)	260 (1)	798 (3)
Rhode Island	8	63–100	22,637	10,653 (47)	4,907 (22)	2,120 (9)	388 (2)	55 (—)	9 (—)	241 (1)	4,228 (19)
South Carolina	23	70–91	63,976	4,705 (7)	15,311 (24)	36,609 (57)	686 (1)	124 (—)	12 (—)	321 (1)	6,097 (10)
South Dakota	4	50–100	5,966	1,194 (20)	3,536 (59)	67 (1)	223 (4)	283 (5)	14 (—)	16 (—)	631 (11)
Tennessee	29	59–83	85,712	8,496 (10)	53,673 (63)	8,411 (10)	430 (1)	63 (—)	53 (—)	1,018 (1)	13,549 (16)
Texas	72	69–85	353,081	109,844 (31)	55,515 (16)	42,531 (12)	10,324 (3)	766 (—)	170 (—)	1,510 (—)	132,353 (37)
U.S. Virgin Islands	2	0–50	365	100 (27)	45 (12)	200 (55)	0 (—)	0 (—)	0 (—)	20 (5)	0 (—)
Utah	13	62–92	13,870	5,451 (39)	4,663 (34)	209 (2)	65 (—)	622 (4)	26 (—)	125 (1)	2,671 (19)
Vermont	11	64–100	5,017	143 (3)	4,415 (88)	42 (1)	41 (1)	16 (—)	0 (—)	10 (—)	348 (7)
Virginia	26	65–88	35,346	8,234 (23)	15,027 (43)	6,979 (20)	381 (1)	140 (—)	4 (—)	211 (1)	4,350 (12)
Washington	27	70–89	98,481	34,877 (35)	30,972 (31)	3,647 (4)	3,844 (4)	1,317 (1)	240 (—)	1,664 (2)	20,566 (21)
West Virginia	28	61–89	47,084	2,864 (6)	39,019 (83)	1,748 (4)	48 (—)	18 (—)	12 (—)	221 (—)	3,153 (7)
Wisconsin	16	69–100	23,098	10,532 (46)	4,309 (19)	1,962 (8)	153 (1)	150 (1)	15 (—)	33 (—)	5,932 (26)
Wyoming	6	33–83	1,304	559 (43)	595 (46)	14 (1)	6 (—)	25 (2)	1 (—)	22 (2)	82 (6)

**Abbreviations**: AI/AN = American Indian/Alaska Native; COVID-19 = coronavirus disease 2019; FQHC = Federally Qualified Health Center; NH/PI = Native Hawaiian/Other Pacific Islander; NH = non-Hispanic.

* SARS-CoV-2 viral tests include polymerase chain reaction and antigen tests.

^†^ The Health Resources Services Administration (HRSA) funds health centers in all 50 states, the District of Columbia, and the following U.S. territories and freely associated states: American Samoa, Federated States of Micronesia, Guam, Northern Mariana Islands, Marshall Islands, Puerto Rico, and U.S. Virgin Islands.

^§^ In June 2020, the number of HRSA-fund health centers was 1,382. By September, the number of HRSA-funded centers decreased to 1,376, by three in California and by one each in Massachusetts, Missouri, New Jersey, and Puerto Rico. By October, the number of Puerto Rico’s HRSA-funded health centers increased by one.

^¶^ FQHCs receive HRSA Health Center Program federal grant funding to improve the health of underserved populations

** The weekly response rate was calculated using the number of health centers that responded to the survey as the numerator and number of current HRSA-funded health centers as the denominator. The response range represents the lowest response rate and the highest response rate nationally and by state during June 5–October 2, 2020.

^††^ Data for the number of persons receiving testing or who had positive test results are aggregated by health center before submission and cannot be deduplicated, which might inflate or misrepresent the number of patients who received testing or who had positive test results.

**TABLE 2:** Positive viral tests for SARS-CoV-2,* by race/ethnicity and jurisdiction^†^ — Health Center COVID-19 Survey, United States, June 5–October 2, 2020

Jurisdiction	Total positive^§^	Race/Ethnicity, no. positive (row %)							
		Hispanic/Latino	White, NH	Black, NH	Asian, NH	AI/AN, NH	NH/PI, NH	Multiracial	Unreported
**United States**	**308,780**	**140,462 (45)**	**59,959 (19)**	**38,385 (12)**	**6,792 (2)**	**1,262 (—)**	**473 (—)**	**2,378 (1)**	**57,208 (19)**
Alabama	5,097	710 (14)	1,469 (29)	2,296 (45)	23 (—)	14 (—)	0 (—)	32 (1)	542 (11)
Alaska	961	81 (8)	167 (17)	31 (3)	18 (2)	253 (26)	0 (—)	7 (1)	399 (42)
American Samoa	0	0 (—)	0 (—)	0 (—)	0 (—)	0 (—)	0 (—)	0 (—)	0 (—)
Arizona	8,297	4,719 (57)	1,857 (22)	243 (3)	25 (—)	178 (2)	9 (—)	70 (1)	1,190 (14)
Arkansas	5,946	1,808 (30)	1,738 (29)	883 (15)	14 (—)	13 (—)	7 (—)	10 (—)	886 (15)
California	46,113	33,310 (72)	4,075 (9)	2,458 (5)	997 (2)	76 (—)	63 (—)	572 (1)	4,470 (10)
Colorado	4,656	3,234 (69)	721 (15)	142 (3)	49 (1)	14 (—)	3 (—)	10 (—)	480 (10)
Connecticut	3,904	2,032 (52)	221 (6)	229 (6)	33 (1)	2 (—)	1 (—)	3 (—)	1,379 (35)
Delaware	244	144 (59)	19 (8)	71 (29)	4 (2)	2 (1)	0 (—)	0 (—)	4 (2)
District of Columbia	1,697	737 (43)	82 (5)	696 (41)	16 (1)	4 (—)	0 (—)	6 (—)	154 (9)
Federated States of Micronesia	0	0 (—)	0 (—)	0 (—)	0 (—)	0 (—)	0 (—)	0 (—)	0 (—)
Florida	43,859	17,913 (41)	3,500 (8)	3,347 (8)	131 (—)	9 (—)	2 (—)	142 (—)	18,802 (43)
Georgia	13,130	2,984 (23)	4,213 (32)	4,204 (32)	69 (1)	6 (—)	2 (—)	48 (—)	1,597 (12)
Guam	633	2 (—)	5 (1)	0 (—)	222 (35)	0 (—)	0 (—)	35 (6)	2 (—)
Hawaii	907	63 (7)	27 (3)	3 (—)	144 (16)	1 (—)	234 (26)	20 (2)	78 (9)
Idaho	2,938	1,185 (40)	991 (34)	119 (4)	55 (2)	94 (3)	18 (1)	9 (—)	467 (16)
Illinois	16,752	6,993 (42)	3,571 (21)	2,074 (12)	151 (1)	16 (—)	1 (—)	55 (—)	3,874 (23)
Indiana	3,274	1,372 (42)	600 (18)	238 (7)	152 (5)	2 (—)	0 (—)	12 (—)	897 (27)
Iowa	3,634	1,011 (28)	1,550 (43)	141 (4)	68 (2)	14 (—)	1 (—)	25 (1)	821 (23)
Kansas	2,810	1,345 (48)	986 (35)	177 (6)	27 (1)	30 (1)	5 (—)	3 (—)	226 (8)
Kentucky	4,191	579 (14)	2,884 (69)	306 (7)	35 (1)	0 (—)	2 (—)	22 (1)	361 (9)
Louisiana	4,922	734 (15)	1,826 (37)	2,088 (42)	29 (1)	11 (—)	0 (—)	37 (1)	191 (4)
Maine	119	28 (24)	42 (35)	43 (36)	0 (—)	0 (—)	0 (—)	1 (1)	5 (4)
Marshall Islands	0	0 (—)	0 (—)	0 (—)	0 (—)	0 (—)	0 (—)	0 (—)	0 (—)
Maryland	1,472	763 (52)	148 (10)	410 (28)	24 (2)	13 (1)	1 (—)	19 (1)	93 (6)
Massachusetts	10,029	6,755 (67)	1,167 (12)	696 (7)	312 (3)	1 (—)	1 (—)	50 (—)	1,031 (10)
Michigan	3,931	1,060 (27)	1,455 (37)	460 (12)	65 (2)	9 (—)	2 (—)	48 (1)	828 (21)
Minnesota	2,194	991 (45)	219 (10)	527 (24)	190 (9)	22 (1)	1 (—)	2 (—)	241 (11)
Mississippi	3,412	255 (7)	686 (20)	2,348 (69)	23 (1)	7 (—)	1 (—)	29 (1)	54 (2)
Missouri	5,770	1,319 (23)	2,310 (40)	1,090 (19)	48 (1)	8 (—)	0 (—)	23 (—)	916 (16)
Montana	865	26 (3)	323 (37)	2 (—)	1 (—)	30 (3)	1 (—)	2 (—)	480 (55)
Nebraska	1,749	1,171 (67)	160 (9)	138 (8)	131 (7)	2 (—)	0 (—)	6 (—)	140 (8)
Nevada	2,893	2,029 (70)	325 (11)	121 (4)	127 (4)	7 (—)	21 (1)	14 (—)	245 (8)
New Hampshire	139	91 (65)	32 (23)	8 (6)	4 (3)	0 (—)	0 (—)	0 (—)	4 (3)
New Jersey	3,275	1,080 (33)	266 (8)	287 (9)	40 (1)	0 (—)	0 (—)	16 (—)	1,584 (48)
New Mexico	729	531 (73)	89 (12)	8 (1)	2 (—)	23 (3)	0 (—)	7 (1)	66 (9)
New York	10,697	1,404 (13)	1,382 (13)	1,507 (14)	1,775 (17)	6 (—)	3 (—)	75 (1)	4,528 (42)
North Carolina	8,467	3,922 (46)	1,846 (22)	1,623 (19)	63 (1)	53 (1)	8 (—)	40 (—)	908 (11)
North Dakota	402	13 (3)	173 (43)	49 (12)	17 (4)	8 (2)	0 (—)	0 (—)	142 (35)
Northern Mariana Islands	0	0 (—)	0 (—)	0 (—)	0 (—)	0 (—)	0 (—)	0 (—)	0 (—)
Ohio	4,794	1,014 (21)	1,881 (39)	1,197 (25)	88 (2)	2 (—)	21 (—)	33 (1)	556 (12)
Oklahoma	1,264	500 (40)	419 (33)	70 (6)	14 (1)	46 (4)	1 (—)	10 (1)	197 (16)
Oregon	1,785	1,011 (57)	266 (15)	79 (4)	346 (19)	14 (1)	1 (—)	3 (—)	62 (3)
Palau	0	0 (—)	0 (—)	0 (—)	0 (—)	0 (—)	0 (—)	0 (—)	0 (—)
Pennsylvania	3,573	1,105 (31)	954 (27)	719 (20)	357 (10)	7 (—)	1 (—)	87 (2)	333 (9)
Puerto Rico	2,137	2,095 (98)	5 (—)	5 (—)	0 (—)	0 (—)	0 (—)	0 (—)	32 (1)
Rhode Island	2,808	1,943 (69)	250 (9)	196 (7)	35 (1)	0 (—)	2 (—)	19 (1)	360 (13)
South Carolina	5,399	1,087 (20)	1,075 (20)	2,602 (48)	18 (—)	18 (—)	1 (—)	16 (—)	568 (11)
South Dakota	857	167 (19)	542 (63)	5 (1)	65 (8)	18 (2)	1 (—)	1 (—)	58 (7)
Tennessee	13,014	2,726 (21)	6,116 (47)	1,544 (12)	114 (1)	22 (—)	6 (—)	432 (3)	2,038 (16)
Texas	22,444	15,079 (67)	2,588 (12)	1,725 (8)	397 (2)	47 (—)	2 (—)	180 (1)	2,419 (11)
U.S. Virgin Islands	2	0 (—)	0 (—)	2 (100)	0 (—)	0 (—)	0 (—)	0 (—)	0 (—)
Utah	1,597	1,099 (69)	284 (18)	20 (1)	6 (—)	48 (3)	1 (—)	17 (1)	116 (7)
Vermont	35	2 (6)	20 (57)	4 (11)	5 (14)	0 (—)	0 (—)	0 (—)	4 (11)
Virginia	3,966	1,684 (42)	1,272 (32)	633 (16)	23 (1)	6 (—)	0 (—)	17 (—)	331 (8)
Washington	9,601	6,115 (64)	1,295 (13)	265 (3)	219 (2)	92 (1)	39 (—)	95 (1)	1,289 (13)
West Virginia	1,792	249 (14)	1,363 (76)	78 (4)	2 (—)	0 (—)	0 (—)	8 (—)	92 (5)
Wisconsin	3,381	2,090 (62)	418 (12)	175 (5)	19 (1)	6 (—)	10 (—)	4 (—)	650 (19)
Wyoming	223	102 (46)	86 (39)	3 (1)	0 (—)	8 (4)	0 (—)	6 (3)	18 (8)

**Abbreviations:** AI/AN = American Indian/Alaska Native; COVID-19 = coronavirus disease 2019; NH = non-Hispanic; NH/PI = Native Hawaiian/Other Pacific Islander.

* SARS-CoV-2 viral tests include polymerase chain reaction and antigen tests.

^†^ The Health Resources Services Administration funds health centers in all 50 states, the District of Columbia, and the following U.S. territories and freely associated states: American Samoa, Federated States of Micronesia, Guam, Northern Mariana Islands, Marshall Islands, Puerto Rico, and U.S. Virgin Islands.

^§^ Data for the number of persons receiving testing or who had positive test results are aggregated by health center before submission and cannot be deduplicated, which might inflate or misrepresent the number of patients who received testing or who had positive test results.

## Discussion

Health centers’ efforts to increase testing for SARS-CoV-2 are an important mitigation strategy to reach racial and ethnic minority groups at increased risk for COVID-19. Published state and national data indicate that racial and ethnic minority groups might be more likely to become infected with SARS-CoV-2, experience more severe COVID-19–associated illness, and have higher risk for death from COVID-19 (*2*–*7*). This study contributes to understanding current health center testing patterns and areas for improvement. Long-standing social inequalities and health disparities among racial and ethnic minority groups likely result from a multitude of factors that lead to increased risk for getting ill and dying of COVID-19, including discrimination,**** limited health care access and utilization, occupation, housing, and educational and income gaps.^††††^ Further, these factors might contribute to other risk factors for severe disease and death, including limited health care access, underlying medical conditions, and higher levels of environmental exposure. The factors contributing to disparities likely vary widely within and among groups, depending on geographic location and other contextual factors.

Health centers have a long-standing commitment to meeting the primary care needs of their communities (*8*). HRSA has awarded funding^§§§§^to support health centers to purchase, administer, and expand capacity for COVID-19 testing and response-related activities, which has enabled health centers to maintain or increase their staffing levels, conduct training, purchase personal protective equipment, and administer tests. Health center services, including testing, contact tracing, isolation, providing health care, and aiding recovery from the impact of unintended negative consequences^¶¶¶¶^ of mitigation strategies, have increased the capacity of health centers to reach populations at increased risk for COVID-19 as well as access to testing and care.*****

A recent analysis of SARS-CoV-2 testing in a multistate network of health centers during the first weeks of the COVID-19 pandemic reported small racial differences in testing and positivity rates; however, larger differences were identified by ethnicity, preferred language, and insurance status, underscoring health centers’ unique position for serving racial and ethnic minority groups and addressing the ongoing need for targeted, language-concordant testing strategies (*9*). The results of this analysis indicate that health centers have afforded racial and ethnic minority populations access to SARS-CoV-2 testing during the COVID-19 pandemic and that these populations were at increased risk for COVID-19, given the large percentage of positive test results. White and Hispanic patients each accounted for 29% of tests performed; however, only 19% of positive test results were among White persons who received testing, whereas 61% were among racial and ethnic minority groups, with the largest percentage of positive test results (45%) among Hispanic patients. Twenty-six states and Puerto Rico reported >40% of positive tests among persons of Hispanic ethnicity with 1.5% of all Hispanic patients receiving testing at Puerto Rican health centers.

The findings in this report are subject to at least five limitations. First, the data used in this analysis are based on responses from health centers that voluntarily reported data to the Health Center COVID-19 Survey and might not be representative of all health centers in the United States, its territories, and freely associated states. Second, data represent a date range of information provided by health centers specified by weekly reporting date. Summary information across report dates is not comparable because of differences in health center responses for a given report date. Third, race and ethnicity data were missing for approximately 22% of patients who received testing and 19% of patients who had positive test results. Fourth, the reported number of patients tested each week does not fully represent the same patients included in the reported number with positive test results that week because of a lag between the date the specimen is collected and the availability of test results. Therefore, positivity cannot be inferred by dividing the number of patients who received positive test results by the number receiving testing. Finally, data for the number of persons with testing or positive results are aggregated by health centers before submission and cannot be deduplicated, which might inflate or misrepresent the number of patients receiving testing or positive test results.

Health centers are an integral component of health systems designed to address structural inequities (*10*). During the COVID-19 public health emergency, health centers have played an important role in providing access to testing in communities disproportionately affected by COVID-19. Health centers’ ability to reach populations at higher risk for SARS-CoV-2 infection might reduce COVID-19 transmission by identifying cases and supporting public health contact tracing and isolation among populations they serve.

**Summary**

**What is already known about this topic?**

Long-standing social inequities and health disparities have resulted in increased risk for COVID-19 infection, severe illness, and death among racial and ethnic minority populations.

**What is added by this report?**

Health centers have provided racial and ethnic minority populations access to SARS-CoV-2 testing. Improving health centers’ ability to reach groups at increased risk for COVID-19 might reduce transmission by identifying cases and supporting contact tracing and isolation.

**What are the implications for public health practice?**

Efforts to improve coordination of COVID-19 response-related activities between state and local public health departments and HRSA-funded health centers can increase access to testing and follow-up care for populations at increased risk for COVID-19.

## Footnotes

* Based on community needs, health centers offer medical, dental, vision, behavioral health, and enabling services.

† https://bphc.hrsa.gov/sites/default/files/bphc/about/healthcenterfactsheet.pdf.

§ Follow up services could include support for public health contact tracing, case investigation, case management, reporting, and isolation/quarantine.

¶ The term “health center” is used to include both Federally Qualified Health Centers (FQHCs) and Health Center Program Look-Alikes (i.e., a health center that meets all Health Center Program requirements but does not receive federal award funding). During COVID-19, HRSA provided one-time COVID-19 funding to FQHCs and Health Center Look-Alikes to purchase, administer, and expand capacity for testing to monitor and support COVID-19 testing and response related activities. Data from FQHCs are included for this analysis.

** https://data.hrsa.gov/tools/shortage-area/mua-find.

†† Areas where economic, geographic, or cultural barriers limit access to affordable health care services.

§§ https://bphc.hrsa.gov/emergency-response/coronavirus-health-center-data/.

¶¶ In June 2020, there were 1,382 HRSA-funded health centers; by September 2020, there were 1,376.

*** In April 2020, HRSA began administering the voluntary weekly Health Center COVID-19 Survey. On June 5, 2020, survey questions related to testing were modified to disaggregate between virus detection (polymerase chain reaction, antigen) and antibody detection (serology). Before June 5, 2020, data from survey questions related to testing might be reflective of COVID-19 tests for both virus and antibody detection.

††† HRSA funds health centers in DC and the following U.S. territories and freely associated states: American Samoa, Federated States of Micronesia, Guam, Northern Mariana Islands, Marshall Islands, Puerto Rico, and U.S. Virgin Islands.

§§§ Patients who reported Hispanic/Latino ethnicity were classified as Hispanic/Latino, regardless of race.

¶¶¶ https://data.census.gov/cedsci/profile?g=0100000US.

**** Discrimination, which includes racism, can lead to chronic and toxic stress and shapes social and economic factors that put some people from racial and ethnic minority groups at increased risk for COVID-19.

†††† https://www.cdc.gov/coronavirus/2019-ncov/community/health-equity/race-ethnicity.html.

§§§§ To date, in 2020, HRSA has awarded approximately $2 billion through three rounds of funding to health centers: 1) March 24: $100 million (https://www.hhs.gov/about/news/2020/03/24/hhs-awards-100-million-to-health-centers-for-covid-19-response.html); 2) April 8:$1.3 billion (https://www.hhs.gov/about/news/2020/04/08/hhs-awards-billion-to-health-centers-in-historic-covid19-response.html); and 3) May 7: $583 million (https://www.hhs.gov/about/news/2020/05/07/hhs-awards-more-than-half-billion-across-the-nation-to-expand-covid19-testing.html).

¶¶¶¶ Potential unintended negative consequences include loss of health insurance; food, housing, and income insecurity; mental health concerns; substance use; and violence resulting from social isolation, financial stress, and anxiety.

***** https://www.cdc.gov/coronavirus/2019-ncov/community/health-equity/cdc-strategy.html.
